# Early cerebral volume reductions and their associations with reduced lupus disease activity in patients with newly-diagnosed systemic lupus erythematosus

**DOI:** 10.1038/srep22231

**Published:** 2016-03-01

**Authors:** Anselm Mak, Roger Chun-Man Ho, Han-Ying Tng, Hui Li Koh, Joanna Su Xian Chong, Juan Zhou

**Affiliations:** 1 Department of Medicine, Yong Loo Lin School of Medicine, National University of Singapore; 2 Department of Psychological Medicine, Yong Loo Lin School of Medicine, National University of Singapore; 3 Division of Rheumatology, University Medicine Cluster, National University Health System, Singapore; 4 Department of Psychological Medicine, University Medicine Cluster, National University Health System, Singapore; 5 Center for Cognitive Neuroscience, Neuroscience and Behavioral Disorders Program, Duke-National University of Singapore Graduate Medical School, Singapore; 6 Clinical Imaging Research Centre, the Agency for Science, Technology and Research and National University of Singapore, Singapore

## Abstract

We examined if cerebral volume reduction occurs very early during the course of systemic lupus erythematosus (SLE), and observed prospectively whether gray (GMV) and white matter volumes (WMV) of the brain would improve with lowered SLE disease activity. T1-weighted MRI brain images were obtained from 14 healthy controls (HC) and 14 newly-diagnosed SLE patients within 5 months of diagnosis (S1) and after achieving low disease activity (S2). Whole brain voxel-based morphometry was used to detect differences in the GMV and WMV between SLE patients and HC and those between SLE patients at S1 and S2. SLE patients were found to have lower GMV than HC in the middle cingulate cortex, middle frontal gyrus and right supplementary motor area, and lower WMV in the superior longitudinal fasciculus, cingulum cingulate gyrus and inferior fronto-occipital fasciculus at both S1 and S2. Whole-brain voxel-wise analysis revealed increased GMV chiefly in the prefrontal regions at S2 compared to S1 in SLE patients. The GMV increase in the left superior frontal gyrus was significantly associated with lowered SLE disease activity. In conclusion, GMV and WMV reduced very early in SLE patients. Reduction of SLE disease activity was accompanied by region-specific GMV improvement in the prefrontal regions.

Systemic lupus erythematosus (SLE) is a prototypic autoimmune condition which potentially affects major organ systems including the central nervous system (CNS)^1^,^2^. The recent advent of structural and functional neuroimaging of the brain has been recognized to be promising in detecting, monitoring and possibly predicting lupus-related CNS damage^3^. Amongst the various abnormal neuroimaging features of CNS damage detected by computed tomography (CT) and magnetic resonance imaging (MRI) in patients with SLE, cerebral atrophy is one of the most commonly described abnormalities^4^,^5^,^6^. Cerebral atrophy has its clinical relevance in its association with cognitive dysfunction, one of the commonest manifestations of neuropsychiatric SLE when neuroimaging findings were assessed in tandem with neuropsychological tests^5^,^6^,^7^. Given the current advancement and maturation of sophisticated brain imaging techniques and automated analyses of MRI brain images, detailed delineation of the locations, anatomical layers and connections where cerebral atrophy takes place have been described^8^. Such fine delineation offers a platform to further understand the potential neuropathological mechanisms behind neuropsychiatric manifestations in patients with SLE.

Lupus-related cerebral atrophy can be caused by reduction in the white matter volume (WMV) due to axonal damage or demyelination, and/or reduction in the gray matter volume (GMV) as a result of cortical atrophy^9^. Reductions of WMV and GMV have been shown in patients with SLE in a number of structural imaging studies and have been generally believed to be attributed by long disease duration, long-term glucocorticoid use and accumulation of lupus-related damage^10^,^11^,^12^,^13^. However, it is evident that cerebral volume loss can occur in patients with short disease duration and without substantial exposure to long-term glucocorticoids^14^,^15^. For instance, extensive WM losses have been found in young patients with juvenile SLE^14^. More intriguingly, with the use of cerebral CT and ultrasound, WM attenuation was noted in 5 out of 10 infants with neonatal lupus who were born from lupus mothers^15^. A point of note is, none of these infants developed subsequent neurological issues and their development was uneventful^15^. Recently, a cross-sectional structural MRI study showed that generalized cerebral volume losses in both the white matter (WM) and gray matter (GM) were evident in adult SLE patients as early as within 9 months after the diagnosis^16^. In an attempt to detect potentially inflammation-triggered volume loss in the brains of lupus patients, a diffuse increase in the uptake of 18-fluoro-D-gluocose (^18^FDG) was mainly found in the WM of the brains detected by positron emission tomography (PET)/CT scans in newly-diagnosed adult lupus patients, and the increased ^18^FDG uptake in the WM was significantly correlated with overall lupus disease activity in these patients^17^. Interestingly, in the same study, higher lupus disease activity was found to be significantly associated with reduced ^18^FDG uptake chiefly in the GM where the frontal and temporal regions were involved^17^. Taken together, the appearance of cerebral volume loss, the hyper-metabolic WM and hypo-metabolic GM in lupus patients during their early phase of the disease and the absence of neurological and developmental consequences in individuals with neonatal lupus despite radiological evidence of WM attenuation lead to the hypothesis that cerebral volume reductions and inflammation might be reversible when the inciting pathology is removed or controlled early in the disease process.

In this study, first, we hypothesized that the WMV and GMV of patients with newly diagnosed SLE would be lower as compared to age-, gender and intelligent quotient (IQ)-matched healthy controls (HC). In order to exclude confounding factors which may contribute to cerebral volume loss and inflammation other than SLE *per se*, patients who possessed traditional cardiovascular risk factors, anti-cardiolipin antibodies (aCL) and lupus anticoagulant (LAC) were excluded. Second, we aimed to investigate if there would be regional improvement of GMV and WMV after clinically sufficient disease control was achieved in patients with SLE. If so, we further addressed whether the reduction of lupus disease activity during the early stage of disease would correlate with enhanced cerebral volumes in lupus patients.

## Method

### Subjects

Adult patients who fulfilled the American College of Rheumatology (ACR) classification criteria^18^ for SLE and attended the Lupus Clinic of the National University Hospital, Singapore, were recruited for this study (see more details in our previous study^19^). HC were recruited and matched for age (±4 years), gender, education level and IQ for comparison.

Subjects were excluded if they (1) had a history of neurological, psychiatric or cardiovascular disease, (2) experienced symptoms and signs suggestive of neuropsychiatric lupus as defined by the ACR criteria^20^, (3) were clinically diagnosed to be anxious or/and depressed based on the Hospital Anxiety Depression Scale (HADS)^21^,^22^ with a score of ≥8 units^22^ as administered by a psychiatrist and were concurrently or had a past use psychotropic medications. In addition, patients who carried traditional cardiovascular risk factors such as hypertension, hyperlipidaemia and diabetes mellitus, and those who were tested positive for aCL and LAC were also excluded. The IQ of the subjects was measured using the Wechsler Abbreviated Scale of Intelligence (WASI)^23^ by a certified clinical psychologist. Written informed consent was obtained before subjects were recruited. The methods adopted in this study were in accordance with the approved guidelines set by the local ethics committee – the Domain Specific Review Boards, National Healthcare Group, Singapore, which also approved the protocol of this study.

### Clinical assessments

Disease activity of SLE was assessed using the Systemic Lupus Erythematosus Disease Activity Index (SLEDAI) on the day of the MRI scan^24^. As per standard of practice for lupus patients in our hospital, sera were assayed for complement (C3, C4) and anti-dsDNA levels by immunoturbidimetry and enzyme-linked immunoassays (BioRad) respectively in the standard laboratory of the hospital. Fasting glucose levels and lipid profiles were also collected as part of our standard care. All SLE patients received treatments based on the discretion of their attending rheumatologists. Patients recruited for the first scan (S1) were invited to participate in the second scan (S2) if they met the following criteria: (1) SLEDAI <4 and (2) more than 6 months had elapsed from the date of S1. Fulfilment of these criteria was to ensure that patients had sufficiently low lupus disease activity and allow for declaration of potential cerebral lesions as damage if the lesions persisted for more than 6 months when patients underwent the second scan. The clinical and serological assessments were repeated on the day of the second scan. The second MRI scans at S2 were performed after the mean ± SD (range) of 504.92 ± 267.0 (196–1088) days of treatment when all the patients had inactive disease (SLEDAI < 4).

Comparisons of continuous variables between the patients and HC were made by using independent sample t-test or Mann-Whitney U test where appropriate. To compare the change of clinical parameters within the SLE group between S1 and S2, paired t-test or Wilcoxon signed rank test was used where appropriate. A 2-tailed p-value of <0.05 was considered statistically significant. All statistical analyses were performed using SPSS version 21 (SPSS 2012).

### Image acquisition

All subjects underwent two MRI scans (S1 and S2) on a 1.5-Tesla Siemens Symphony MRI scanner (Siemens, Erlangen, Germany) at the National University Hospital, Singapore. A high resolution T1-weighted MPRAGE (coronal acquisition, 158 slices, matrix size = 256 × 256, voxel size = 1.0 × 1.0 × 1.0 mm^3^, TE = 2.11 ms, TR = 1840 ms, TI = ms, flip angle = 90°, TS = 2 ms, field of view 256 × 256 mm^2^) was acquired from all subjects.

### Image analysis

An optimized voxel-based morphometry (VBM) protocol^25^ using SPM8 (University College London 2014)^26^ was applied in the image analysis. The subject-level GMV probability maps were derived from T1-structural images following a previously described approach^27^ which included (1) segmentation of individual T1-weighed images into GM, WM and cerebrospinal fluid (CSF), (2) creation of a study-specific template using non-linear DARTEL registration^28^, (3) registration for each GM/WM probability maps to the customized template in MNI space and performing tissue segmentation, (4) modulation by multiplying voxel values by the Jacobian determinants derived from the spatial normalization step and (5) application of smoothing on the normalized GM maps by a 8 mm isotropic Gaussian kernel.

### Between-group statistical analyses on GM and WM volumes

To examine changes in GMV (and WMV) between SLE patients and HC, whole brain voxel-wise two-sample t-tests on subject-level GMV (and WMV) probabilistic maps between (1) HC and SLE patients at S1, and (2) HC and SLE patients at S2 after disease control were performed, with total intracranial volume as the nuisance variable. GM regions with group differences were reported by thresholding at p < 0.01 family-wise error (FWE) corrected, with minimum cluster size of 50 voxels. WM regions with group differences were reported by thresholding at p < 0.05 FWE corrected, with minimum cluster size of 50 voxels. All analyses were constrained to GM (WM) region mask derived from the study-specific GM (WM) template thresholded by a probability of 0.5.

### Correlation between brain structures and disease activity of SLE

To examine GMV and WMV changes within the SLE group between S1 and S2, whole brain voxel-wise paired t-tests between subject-level GMV (WMV) probabilistic maps in SLE patients at S1 and S2 was performed at p < 0.001 with minimum cluster size of 980 voxels.

To examine the relationships between the changes in GMV and WMV (S2 minus S1) and SLE disease activity as measured by the SLEDAI, we extracted the subject-level mean GMV (and WMV) of the identified regions of interest (ROI) (from the paired t-tests between S1 and S2) from the modulated and smoothed GMV (and WMV) probabilistic maps of SLE patients at S1 and S2 using the Marsbar toolbox for SPM8^29^. The changes in GMV (and WMV) between S1 and S2 were calculated for each ROI. Spearman correlations were then performed between the changes in SLEDAI and the changes in GMV (and WMV) for each ROI in S2 compared to S1, thresholded at p < 0.05.

## Results

### Clinical parameters and treatment

Fourteen SLE patients and 14 matched HC were recruited in this study. Their demographic and clinical information is summarized in Table 1. The majority of lupus patients (12 out of 14) had active SLE (SLEDAI > 4) at baseline (S1) as evidenced by their low mean ± standard deviation (SD) serum C3 and C4, and high anti-dsDNA levels (Table 1). The mean ± SD (range) interval between the diagnosis of SLE and first MR scan was 36.86 ± 35.5 (8–143) days.

At S2, there were significant improvement in the SLEDAI and reduction of daily prednisolone dose (Table 1). The mean ± SD cumulative dose of prednisolone was 1.76 ± 2.10 grams.

### GMV loss in SLE patients

**Figure 1:**
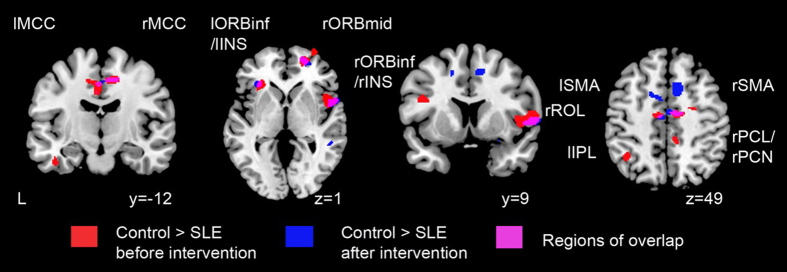
Gray matter volume changes in SLE patients before and after sufficient immunosuppressive treatment and attaining low lupus disease activity as compared to healthy controls. Areas of reduced gray matter volume (GMV) in SLE patients at diagnosis (in red) and after attaining low lupus disease activity (in blue) compared with controls are presented. Regions of overlap are highlighted in magenta, including bilateral MCC, right ROL, bilateral ORBinf, right ORBmid, bilateral INS, and right SMA. Results were thresholded at p < 0.01 family-wise error with minimum cluster size of 50 voxels. Abbreviations: MCC = middle cingulate cortex, ROL = rolandic operculum, ORBinf = inferior frontal gyrus, orbital part, ORBmid = middle frontal gyrus, orbital part, INS = insula, SMA = supplementary motor area, L = left side, l = left, r = right.

Overall, lupus patients had lower GMV mainly in the frontal regions as compared to HC at both S1 and S2, including bilateral middle cingulate cortex, right rolandic operculum, and bilateral middle frontal gyrus orbital part (Fig. 1, regions highlighted in violet; Table 2 and supplementary Fig. 1). Moreover, at S1, lupus patients had lower GMV in bilateral temporal pole, left inferior parietal gyrus, left lingual gyrus, left cerebellum, left insula, bilateral inferior frontal gyrus opercular part and left precentral gyrus as compared to controls (Fig. 1, regions highlighted in red). At S2, additional regions with lower GMV in the cuneus/precuneus, middle temporal gyrus, paracentral lobule/precuneus in the right hemisphere and bilateral supplementary motor area/middle cingulate cortex were noted in the lupus group (Fig. 1, regions highlighted in blue; Table 2). There was no greater GMV in SLE patients as compared to HC at both time points.

### WMV loss in SLE patients

**Figure 2:**
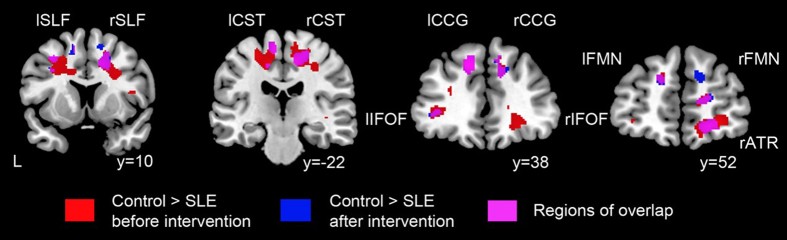
White matter volume changes in SLE patients before and after sufficient immunosuppressive treatment and attaining low lupus disease activity as compared to healthy controls. Areas of reduced white matter volume (WMV) in SLE patients at diagnosis (in red) and after attaining low lupus disease activity (in blue) compared with controls. Regions of overlap are highlighted in magenta, including bilateral SLF, bilateral CST, bilateral CCG, bilateral IFOF, and bilateral FMN, right ATR. Results were thresholded at p < 0.05 family-wise error with minimum cluster size of 50 voxels. Abbreviations: SLF = superior longitudinal fasciculus, CST = corticospinal tract, CCG = cingulum cingulate gyrus, IFOF = inferior fronto-occipital fasciculus, FMN = forceps minor, ATR = anterior thalamic radiation, L = left side, l = left, r = right.

Overall, lupus patients had lower WMV in bilateral superior longitudinal fasciculus, corticospinal tract, cingulum cingulate gyrus and inferior fronto-occipital fasciculus as compared to HC at both S1 and S2 (Fig. 2, Table 3 for more details). In addition, at S1, lupus patients had lower WMV in bilateral forceps major, right inferior longitudinal fasciculus and bilateral ucinate fasciculus as compared to controls (Table 3 for more details). At S2, no additional regions with lower WMV compared to controls was identified. There was no increased WMV in SLE patients as compared to HC at both time points.

### Correlation between brain volumes and disease activity of SLE

**Figure 3:**
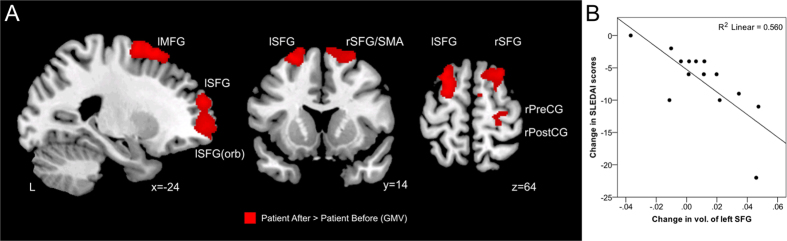
Relationship between changes in gray matter volume and disease activity in SLE patients after sufficient disease control. (**A**) Areas of improvement of gray matter volume (GMV) in SLE patients after disease control included bilateral superior frontal gyrus, left middle frontal gyrus, right supplementary motor area, right precentral gyrus and right postcentral gyrus (p < 0.001 uncorrected with minimum cluster size of 980 voxels). (**B**) Scatterplot of significant Spearman correlation between changes in gray matter volume and SLEDAI after attainment of low SLE disease activity in SLE patients. Abbreviations: MFG = middle frontal gyrus, SFG = superior frontal gyrus, SFG(orb) = superior frontal gyrus (orbital part), SMA = supplementary motor area, PreCG = precentral gyrus, PostCG = postcentral gyrus, SFG = superior frontal gyrus, SLEDAI = Systemic Lupus Erythematosus Disease Activity Index, L = left side, l = left, r = right.

Whole-brain voxel-wise analysis of the SLE patients revealed increased GMV in bilateral superior frontal gyrus, left middle frontal gyrus, right superior supplementary motor area, and right pre/post central gyrus in S2 compared to S1 (p < 0.001 uncorrected with minimum cluster size of 980 voxels) (Fig. 3A, Table 4). Among the five regions, the increased GMV in the left superior frontal gyrus was found to be significantly correlated to the reduction of SLEDAI amongst lupus patients corrected for multiple comparisons p < 0.05 (r^2^ = 0.560, p = 0.002) (Fig. 3B). No change in WMV was detected between S1 and S2.

## Discussion

In this study, we demonstrated that after a mean of 37 days (range 8–143 days) following the diagnosis of SLE, lower GMV and WMV of the brain were evident on MRI in lupus patients who were negative for cardiovascular risk factors, as compared to HC matched for age, gender and IQ. Whole-brain analysis found higher GMV mainly in prefrontal areas after sufficient disease control (SLEDAI < 4) amongst the SLE patients. In addition, there was a significant correlation between reduction of SLE disease activity and increase in GMV in the left superior frontal gyrus, a region crucial in high-level cognitive and executive functioning^30^.

Based on previous structural brain imaging studies, loss of cerebral volume in lupus patients has long been conceptualized to be associated with factors such as long disease duration^10^,^11^, cumulative glucocorticoid use^11^,^12^, the presence of antiphospholipid antibodies^11^,^31^, hypertension^31^, hyperlipidaemia^32^ and lupus-related disease damage^13^. Recently, a cross-sectional study led by Petri *et al.* challenged such concept by demonstrating that cerebral volume loss was already evident in patients with newly-diagnosed SLE within 9 months of diagnosis and more active disease was associated with more focal brain lesions^16^. In our current study, we concurred with Petri *et al.*^16^ by demonstrating that lower cerebral volume was readily detectable in patients early in their courses of SLE. In addition, when compared to healthy subjects, lower GMV and WMV was found to be present much earlier in our SLE patients than those of Petri *et al*. (mean of ~37 days after diagnosis of SLE in our study compared to that of 9 months in Petri *et al.*’s study).

The underlying mechanism of lower cerebral volumes in our lupus patients deserve further discussion. It is currently proposed that the reduction of cerebral volume during the early course of SLE is inflammation driven^17^. In the MRL/lpr murine model which is characterized by early spontaneous development of antinuclear and anti-dsDNA antibodies and subsequent immune-complex glomerulonephritis at the age between 12 and 16 weeks, CNS inflammation is evident before the development of florid lupus-like phenotypes^33^. In human lupus, CNS inflammation was evident as early as within 9 months of the diagnosis of SLE, as demonstrated by the diffuse increase in ^18^FDG uptakes in PET/CT scans in the WM in lupus patients without neuropsychiatric symptoms^17^. Furthermore, the diffusely increased ^18^FDG uptakes in the WM was significantly associated with the increase in the SLEDAI score, suggesting that the hyper-metabolic changes in the WM was associated with lupus-related active inflammation. In the current study, the occurrence of lower WMV as compared to HC in our newly-diagnosed SLE patients implies that CNS inflammation has probably been taking place very early at, or even prior to the diagnosis of SLE. An interesting phenomenon is, in contrast to the WM where hyper-metabolism takes place in lupus patients with active disease, the increase of SLEDAI was shown to be associated with decreased regional ^18^FDG uptakes in the GM^17^. The hypo-metabolic GM mainly involved the prefrontal regions which included the precentral gyrus, postcentral gyrus and medial frontal gyrus^17^. Mechanistically, the coexistence of diffuse WM hyper-metabolism and regional GM hypo-metabolism is best explained by diaschisis, a phenomenon in which diffuse loss of structural and/or functional integrity of the WM triggers cortical functional decline in regions connected to the compromised WM^34^. In the setting of active SLE, WM inflammation during the initial phase of active disease might potentially inflict injury to GM areas which receive fibre tracts from the inflamed WM, leading to regional loss of GMV through diaschisis. Indeed, apart from the lower GMV and WMV in our patients with newly-diagnosed SLE, our prospective data hitherto showed that the areas where GMV significantly improved after adequate suppression of SLE activity (SLEDAI < 4) in our patients were located in the prefrontal regions which are functionally important in executive functioning^30^. In addition, we found that the improvement of the volume of the left superior frontal gyrus was significantly associated with the reduction of SLEDAI amongst the newly-diagnosed SLE patients after the mean of 17 months of immunosuppressive therapy. With the use of functional MRI coupled with N-back working memory test in a lesion study, the left superior frontal gyrus has recently been discovered to be crucial in mediating high-level cognitive function and executive processing^30^. In our recent functional MRI study in which the same group of SLE patients were challenged with a cognitive-shifting task at S1, the brain activity of the Brodmann area (BA) 10, the region where the left superior frontal gyrus is located, was significantly activated when compared to healthy subjects^19^. Such compensatory activation as a consequence of augmentation of blood flow in the vicinity of BA10 attempted to maintain the performance of cognitive-shifting task that required executive processing, with an aim to compensate partially for the overall inferior strategic planning skill amongst the patients with SLE^19^. Thus, the association between the reduction of lupus disease activity and the higher volume of the left superior frontal gyrus found in our study may imply a beneficial impact of lowering SLE disease activity early in the course of the disease on enhancement of executive functioning. Besides the left superior frontal gyrus, we found an increase in GMV in the right supplementary motor area which was close to the superior frontal gyrus amongst the lupus patients after attaining low SLE disease activity. In contrast, the GMV of different parts of bilateral supplementary motor areas, which were mostly inferior and covering part of the mid-cingulate cortex, was lower in the SLE patients compared to those of the healthy controls at S2. Thus, the fact that the regions where higher GMV were found after disease control did not overlap with those with lower GMV in patients at S2 may imply activity-driven structural compensation in other brain regions rather than recovery of the injured regions.

Intuitively, inflammation-induced insults take time to engender loss of cerebral volume. Besides the presence of a time lag period between symptom onset and disease diagnosis of SLE when inflammation-induced cerebral atrophy is taking place, it has been demonstrated that the presence of lupus-related autoantibodies preceded the clinical diagnosis of SLE^35^. Amongst all, antinuclear, anti-dsDNA and anti-Ro antibodies have been thought to be associated with impaired cognitive functioning and diffuse neuropsychiatric SLE^36^,^37^. They can be present in patients for as long as 9 years before the clinical diagnosis of SLE^35^. Perhaps, these antibodies have already triggered a certain degree of CNS inflammation and induced cerebral volume reduction before the development of other clinically-overt lupus manifestations which eventually lead to the diagnosis of SLE. To test this hypothesis, prospective imaging studies in individuals with positive lupus-related autoantibodies who have not yet developed clinical SLE are necessary. Nevertheless, given that the SLE patients in this study had active SLE at baseline and they did not have concurrent cardiovascular and cerebrovascular diseases and the presence of traditional cardiovascular risk factors, aCL and LAC, the contribution of lupus-related inflammation to the reduction of cerebral volume early in the course the disease in our findings should be substantial.

The current study has its limitations. Firstly, the sample size is not large enough. Nevertheless, it should still be noted that the whole-brain voxelwise, highly-sensitive and optimised VBM approach that we adopted with the use of SPM8 was able to delineate regions with significantly lower WMV and GMV in patients with SLE when compared to a carefully matched group of HC at conservative thresholds. Secondly, although the mean time between the first scan and diagnosis was only about 37 days, it can be argued that the lag time between symptom onset and the diagnosis of SLE was potentially sufficient to expose our patients’ brains to inflammation-induced cerebral volume loss. Indeed, most of our lupus patients were referred from the government-based general outpatient clinics geographically close to our hospital and were triaged to attend the Lupus clinic within 6 weeks if the referrals were urgent. Thus, the duration between symptom onset and diagnosis was minimal. Thirdly, we demonstrated higher cerebral volumes after sufficient disease control in a few GM regions that mainly correspond to where normal executive functions are mediated. Unfortunately, we did not perform prospective neuropsychological tests to confirm the phenotypic improvement, although such GMV enhancement was associated with reduced disease activity. Moreover, the observed GMV differences derived from VBM might be confounded by inaccurate tissue segmentation or registration^38^. Although a previous study demonstrated that lupus patients with cognitive dysfunction ascertained by standard neuropsychological tests were not different from those without cognitive dysfunction in white matter changes and ventricle-to-brain ratios, it is still useful to address prospectively the relationship between cognitive impairment and changes of brain volumes in SLE patients^39^. Fourthly, although it can be argued that glucocorticoids might affect cerebral volumes, our analyses did not find significant correlation between prednisolone dose and cerebral volumes at S1 and S2 (data not shown). Lastly, while every effort was made to exclude patients with confounding factors such as those with traditional cardiovascular risks, history of cerebrovascular disease, LAC and aCL, we did not exclude patients who might have other unconventional atherogenic or inflammatory factors including hyperhomocysteinaemia, hyperfibrogenaemia and oxidative stress.

In conclusion, lower volumes in the GM and WM were detectable quite early in patients during their course of SLE. Reduction of SLE disease activity was associated with higher GMV in a number of prefrontal regions. Particularly, the reduction of SLEDAI was found to be significantly associated with higher volume of the left superior frontal gyrus, a cortical region crucial in high-level cognitive and executive functioning. In-depth and prospective validation of our radiological findings by coupling functional brain MRI with cognitive and executive testing in a larger number of patients will be carried out by our team in the coming 3 years with our recently secured national research funding (R-172-000-337-511).

## Additional Information

**How to cite this article**: Mak, A. *et al.* Early cerebral volume reductions and their associations with reduced lupus disease activity in patients with newly-diagnosed systemic lupus erythematosus. *Sci. Rep.* **6**, 22231; doi: 10.1038/srep22231 (2016).

## Supplementary Material

Supplementary Information

## Figures and Tables

**Table 1:** Demographic and clinical characteristics of SLE patients and controls.

	SLE patients atdiagnosis (S1) (N = 14)	SLE patients after diseasecontrol (S2) (N = 14)	Healthy controls(N = 14)	p Value
	Mean (SD)			
Demographics				
Age, years	39.38 (13.9)		34.07 (14.4)	NS
Gender, female (%)	12 (85.70)		12 (85.70)	NS
Presenting clinical features of SLE†				
Rash		10 (71.4)		
Arthritis		10 (71.4)		
Raynaud’s phenomenon		1 (7.14)		
Proliferative glomerulonephritis		6 (42.9)		
Serositis Clinical parameters		1 (71.4)		
SLEDAI	9.93 (5.7)	2.64 (1.4)	—	<0.001
C3, mg/dL	70.46 (25.0)	89.08 (17.8)	—	0.009
C4, mg/dL	15.42 (10.9)	20.67 (8.8)	—	0.020
Anti-dsDNA, IU	132.50 (111.3)	83.42 (94.0)	—	0.064
Prednisolone, mg/day	15.32 (18.4)	3.82 (3.5)	—	0.023
Cumulative prednisolone dose, gm	1.76 (2.1)	—	—	
Medications, number (%)				
Prednisolone	14 (100)	11 (78.6)	—	0.222
Hydroxychloroquine	8 (57.1)	10 (71.4)	—	0.695
Azathioprine	3 (21.4)	8 (57.1)	—	0.120
Cyclophosphamide	4 (28.6)	0 (0)	—	0.098
Mycophenolate mofetil	0 (0)	1 (7.1)	—	1.000
Methotrexate	1 (7.1)	1 (7.1)	—	1.000
Leflunomide	0 (0)	1 (7.1)	—	1.000
Serum CVS risk factors†				
HDL-c, mmol/L	1.50 (0.4)	—	—	
LDL-c, mmol/L	2.94 (0.6)	—	—	
TG, mmol/L	1.21 (0.6)	—	—	
HbA1c, %	5.4 (0.5)	—	—	
Cardiac Index	3.46 (0.9)	—	—	
Time between SLE diagnosis andfirst scan, days (range)	36.86 ± 35.5 (8–143)			
Interval between S1 and S2,days (range)	504.92 ± 267.0 (196–1088)			
WASI Scores				
Verbal	96.23 (15.59)	99.00(14.43)	100.43 (11.01)	0.424
Performance	101.50 (19.84)	105.21 (18.98)	104.86 (11.60)	0.589
Full IQ	99.31 (17.10)	104.86 (11.60)	103.14 (11.22)	0.494

^†^A single patient could present with more than one clinical manifestations of SLE. Abbreviations: S1 = time of first MRI scan at diagnosis, S2 = time of the second MRI scan after disease control, SD = standard deviation, NS = not significant, SLEDAI = Systemic Lupus Erythematosus Disease Activity Index, IU = International Unit, CVS = cardiovascular, HDL-c = high-density lipoprotein, LDL = low-density lipoprotein, TG = triglyceride, HbA1c = glycated haemoglobin, WASI = Wechsler Abbreviated Scale of Intelligence.

**Table 2:** Brain regions showing reduced grey matter volume in SLE patients when compared to healthy controls.

Brain regions	L/R	At diagnosis (S1)					After attaining low SLE activity (S2)				
		MNI Coordinates (mm)			Analysis		MNI Coordinates (mm)			Analysis	
		x	y	z	t-stats	size	x	y	z	t-stats	size
ORBinf/TPOmid	L	−30	21	−35	6.56	177	−26	18	−24	6.50	70
INS/ORBinf	R	28	15	−17	6.64	255	36	21	−20	7.66	996
IFGtri/INS	L	−27	30	−2	6.83	203	−32	30	−3	6.71	146
ITG	L	−42	−12	−29	6.96	119	−56	−67	−18	6.50	54
MCC	L	12	−12	49	7.82	443	14	−10	51	7.27	436
MFG/SFG	R	20	53	3	7.67	231	21	53	3	7.31	180
ORBmid	R	22	68	−11	7.07	611	22	68	−12	6.91	97
ROL/IFGoperc	R	57	9	3	8.33	729	36	21	−20	7.66	996
LING	L	−16	−57	−8	6.55	109	—	—	—	—	—
CB	L	−20	−72	−35	6.55	224	—	—	—	—	—
IFGoperc/PreCG	L	−38	5	22	7.39	249	—	—	—	—	—
IPL	L	−39	−52	46	7.38	171	—	—	—	—	—
TPO	R	46	27	−23	6.67	140	—	—	—	—	—
MCC	R	9	−36	43	6.83	148					
PreCG	L	−21	−30	55	7.16	120					
SFG	R	22	−6	51	6.67	61					
PCL/PCUN	R	—	—	—	—	—	10	−39	57	6.92	120
SMA/MCC	R	—	—	—	—	—	10	14	48	7.28	227
SMA/MCC	L	—	—	—	—	—	14	−10	51	7.27	436
CUN/PCUN	R	—	—	—	—	—	14	−75	37	6.79	150
MTG	R	—	—	—	—	—	49	−58	12	6.86	93

Regions having reduced GMV in SLE patients compared to healthy controls were reported at a height threshold of p < 0.01 family-wise error corrected with the minimum cluster size of 50 voxels (p < 0.05 extent threshold). S1: Regions of Interest (ROIs) having reduced GMV in SLE patients at diagnosis. S2: ROIs having reduced GMV in SLE patients after attaining low SLE disease activity. The t-stats and coordinates (x, y and z in mm) of the peak voxel within each cluster are given. The size of each cluster (number of voxels) are also provided (each voxel equals to 1.5 mm^3^).

Abbreviations: L = left, R = right, ORBinf = inferior frontal gyrus (orbital part), IFGtri = inferior frontal gyrus (triangular part), ITG = inferior temporal gyrus, MCC = middle cingulate cortex, MFG = middle frontal gyrus, ORBmid = middle frontal gyrus (orbital part), INS = insula, ROL = rolandic operculum, SFGdor = superior frontal gyrus (dorsolateral), LING = lingual gyrus, CB = cerebellum, TPO = temporal pole, IFGoperc = inferior frontal gyrus (opercular part), PreCG = precentral gyrus, IPL = inferior parietal, excluding supramarginal and angular gyri, PCL = paracentral lobule, PCUN = precuneus, SMA = supplementary motor area, CUN = cuneus, MTG = middle temporal gyrus.

**Table 3:** Brain regions showing reduced white matter volume in SLE patients when compared to healthy controls.

Brainregions	L/R	At diagnosis (S1)					After attaining low SLE activity (S2)				
		MNI Coordinates (mm)			Analysis		MNI Coordinates (mm)			Analysis	
		x	y	z	t-stats	size	x	y	z	t-stats	size
ATR	L	−14	38	38	6.93	488	−14	38	38	6.20	133
ATR	R	21	56	−9	6.98	549	21	57	12	6.34	238
CST	L	−8	−24	63	6.50	1335	−8	−24	63	6.19	219
CST	R	15	−20	53	6.52	1297	11	−30	66	6.11	733
CCG	L	−12	38	38	6.92	348	−9	38	39	6.28	147
CCG	R	12	11	41	6.27	364	12	14	39	6.31	300
FMN	L/R	−14	38	36	6.81	841	21	59	11	6.45	532
IFOF	L	−12	38	39	6.86	295	−11	38	39	6.23	126
IFOF	R	20	56	−9	6.97	811	20	56	−9	6.28	103
SLF	L	−14	36	39	6.80	1065	−23	−6	51	6.21	221
SLF	R	47	5	14	6.30	433	23	−39	56	6.00	110
FMJ	L/R	14	−93	0	6.69	1110	—	—	—	—	—
ILF	R	42	−57	−2	6.06	209	—	—	—	—	—
UF	L	−14	38	39	6.88	83	—	—	—	—	—
UF	R	23	54	−11	6.45	119	—	—	—	—	—

Regions having reduced WMV in SLE patients compared to healthy controls were reported at the height threshold of p < 0.05 family-wise error corrected with the minimum cluster size of 50 voxels (p < 0.05 extent threshold). S1: Controls versus SLE patients at diagnosis. S2: Controls versus SLE patients after sufficient disease control. The t-stats and coordinates (x, y and z in mm) of the peak voxel within each cluster are given. The size of each cluster (number of voxels) are also provided (each voxel equals to 1.5 mm^3^).

Abbreviations: L = left, R = right. ATR = anterior thalamic radiation, CST = corticospinal tract, CCG = cingulum cingulate gyrus, FMN = forceps minor, IFOF = inferior fronto-occipital fasciculus, SLF = superior longitudinal fasciculus, FMJ = forceps major, ILF = inferior longitudinal fasciculus, UF = ucinate fasciculus.

**Table 4:** Brain regions showing increased gray matter volume (GMV) after treatment.

Brain regions	L/R	Patient After > Patient Before (Gray Matter Volume)					
		MNI Coordinates (mm)			Analysis		
		x	y	z	t-stats	size	p-value
SFG/SFG (orb)	L	−21	56	21	5.04	1771	0.0002264
SFG/MFG	L	−24	18	64	5.01	1330	0.0002387
PreCG/PostCG	R	28	−33	73	4.84	1051	0.00032287
SFG/SMA	R	27	18	64	4.70	1426	0.00041522

Regions having increased GMV in SLE patients were reported at the height threshold of p < 0.001 uncorrected with the minimum cluster size of 980 voxels. Coordinates (x, y and z) are given in mm according to MNI space. The t-stats and p-values of the peak coordinates within each clusters are provided in the table.

Abbreviations: L = left, R = right, SFG = superior frontal gyrus, SFG (orb) = superior frontal gyrus (orbital part), MFG = middle frontal gyrus, SMA = supplementary motor area, PreCG = precentral gyrus, PostCG = postcentral gyrus.
