# Supplementary material for: Early cerebral volume reductions and their associations with reduced lupus disease activity in patients with newly-diagnosed systemic lupus erythematosus
Source: Sci Rep. 2016 Mar 1;6:22231. doi: 10.1038/srep22231 (PMC4772001; doi:10.1038/srep22231)
Supplement: Supplementary Information [file srep22231-s1.pdf]

Early cerebral volume reductions and their associations with reduced lupus disease activity in patients with newly-diagnosed systemic lupus erythematosus

[1, 3]Anselm Mak, [2, 4]Roger Chun-Man Ho, [5]Han-Ying Tng, [5]Hui Li Koh, [5]Joanna Su Xian Chong, [5,6]Juan Zhou

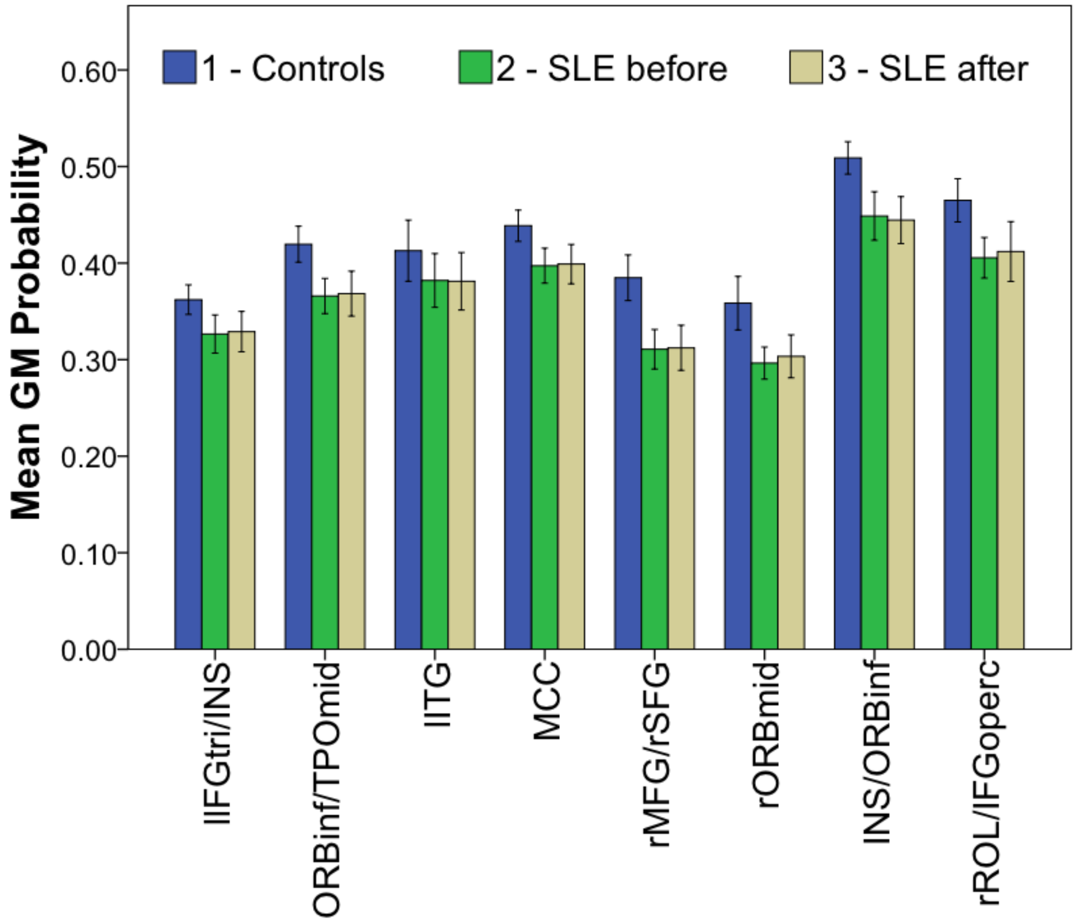

Supplementary Figure 1. Group differences in gray matter (GM) volume. Each bar represents the grey matter volume probability of each cluster (mean and +/- 2 standard error) in controls, lupus group at S1 (before treatment) and S2 (after treatment).

Abbreviations: l = left, r = right, IFGtri = inferior frontal gyrus (triangular part), INS = insula, ORBinf = inferior frontal gyrus (orbital part), TPOmid = middle temporal pole, ITG = inferior temporal gyrus, MCC = middle cingulate cortex, MFG = middle frontal gyrus, SFG = superior frontal gyrus, ORBmid = middle frontal gyrus (orbital part), ROL = rolandic operculum, IFGoperc = inferior frontal gyrus (opercular part).
